# Demographic and Psychosocial Correlates of COVID-19 Vaccination Status among a Statewide Sample in Texas

**DOI:** 10.3390/vaccines11040848

**Published:** 2023-04-15

**Authors:** Justin M. Luningham, Idara N. Akpan, Tanjila Taskin, Sarah Alkhatib, Jamboor K. Vishwanatha, Erika L. Thompson

**Affiliations:** 1 Department of Biostatistics and Epidemiology, School of Public Health, University of North Texas Health Science Center, 3500 Camp Bowie Blvd, Fort Worth, TX 76107, USA; 2 Department of Health Behavior and Health Systems, School of Public Health, University of North Texas Health Science Center, 3500 Camp Bowie Blvd, Fort Worth, TX 76107, USA; 3 Institute for Health Disparities, University of North Texas Health Science Center, Fort Worth, TX 76107, USA

**Keywords:** COVID-19, vaccination, disparities, trust

## Abstract

The COVID-19 pandemic has been a global public health concern since early 2020 and has required local and state-level responses in the United States. There were several Food and Drug Administration (FDA) approved vaccines available for the prevention of COVID-19 as of August 2022, yet not all states have achieved high vaccination coverage. Texas is a particularly unique state with a history of opposing vaccination mandates, as well as a large and ethnically/racially diverse population. This study explored the demographic and psychosocial correlates of COVID-19 vaccinations among a statewide sample in Texas. A quota sample of 1089 individuals was surveyed online from June–July 2022. The primary outcome in this study was COVID-19 vaccination status (fully vaccinated, partially vaccinated, or unvaccinated) and included independent variables related to demographics, COVID-19 infection/vaccine attitudes and beliefs, and challenges related to the COVID-19 pandemic. Hispanic/Latinx individuals were more likely than non-Hispanic White individuals to be partially vaccinated as opposed to unvaccinated. Higher education levels and confidence that the FDA would ensure a safe COVID-19 vaccine were strongly associated with a higher likelihood of being fully vaccinated. In addition, some challenges brought on by the pandemic and concerns about becoming infected or infecting others were associated with a higher likelihood of being partially or fully vaccinated. These findings emphasize the need to further investigate the interaction between individual and contextual factors in improving COVID-19 vaccination rates, especially among vulnerable and disadvantaged populations.

## 1. Introduction

The COVID-19 pandemic has been a global public health concern since early 2020 and has required local and state-level responses in the United States. As of 9 March 2023, there were over 6.6 million confirmed cases of COVID-19 and over 91,000 COVID-19 fatalities in Texas, affecting areas with higher population densities, such as Dallas, Tarrant, Harris, Bexar, and Travis counties [1]. Moreover, disparities in COVID-19 morbidity and mortality have been present in communities with decreased access to care and underrepresented racial/ethnic minorities [2]. Prevention of COVID-19 has required a multi-method approach with evolving evidence, including social distancing, masking, stay-at-home orders, hand washing, and vaccination [3]. Texas was one of the first states to end mask mandates [4], and subsequently, was among the states that prohibited a vaccination mandate [5]. 

There were several Food and Drug Administration approved and Advisory Committee on Immunization Practices recommended vaccines available for the prevention of COVID-19, as of August 2022 [6], yet not all states have achieved high vaccination coverage. According to the Centers for Disease Control and Prevention, Texas ranked 48th in the nation for the proportion of fully vaccinated adults and full population with first booster doses of 40.8% and 37.2%, respectively [7]. A simulation analysis revealed that enhanced COVID-19 vaccinations between December 2020 and August 2021 would have reduced approximately 37,000 hospital admissions and 6,000 deaths in Texas [8].

Texas is a particularly unique state with a history of pushing back against vaccination mandates, vaccine hesitancy, and participation in the antivaccine movement. In addition to the Texas Governor’s executive order to ban required COVID-19 vaccinations, Texas has previously had public and political pushback on a human papillomavirus (HPV) vaccine school-entry requirement in 2007 [9,10]. Texas also has nonmedical exemptions for vaccines required for school entry, which has resulted in several Texas counties being identified as “hotspots” for potential vaccine-preventable disease outbreaks [11]. The use of nonmedical exemptions is also increasing across Texas [12]. While these examples are not directly tied to COVID-19 vaccination, they underscore the level of vaccine hesitancy present in Texas, which may contribute to COVID-19 vaccine hesitancy as well. 

Previous studies have assessed COVID-19 vaccine hesitancy in Texas. A survey prior to COVID-19 vaccine approval found higher vaccine hesitancy among persons who identified as Black or African American and those who reported lower educational attainment and incomes [13]. Similarly, a survey on reproductive-aged women in low-income Texas clinics and pre-vaccine availability found that a majority were hesitant about receiving a COVID-19 vaccine and wanted more information on its safety [14]. A smaller study among patients in a federally qualified health center on the Texas–Mexico border in early 2021 found safety concerns as a motive for non-vaccination [15]. There has since been limited research describing the levels of vaccine hesitancy for COVID-19 vaccinations in Texas since the widespread distribution of vaccines and the availability of boosters. 

Given Texas’ COVID-19 response and history of broad vaccine hesitancy, this study sought to explore the demographic and psychosocial correlates of COVID-19 vaccinations among a statewide representative sample in Texas from June–July 2022. This paper identifies correlates of COVID-19 vaccination status (fully vaccinated, partially vaccinated, and no vaccination) to determine opportunities for improvement across the state. Texas is part of the National Institutes of Health (NIH) Community Engagement Alliance (CEAL) Against COVID-19 Disparities with the mission of using community engagement methods to reduce COVID-19 disparities [16]. Findings from this paper can inform the strategies used by the Texas CEAL initiative and other statewide attempts to reduce the burden of COVID-19. 

## 2. Materials and Methods

### 2.1. Sample

This paper is part of a larger study to assess the COVID-19 vaccination and COVID-19 clinical trial enrollment perspectives in Texas. A quota sample based on the racial/ethnic distribution in Texas was recruited through a Qualtrics Panel. The panel is a double-opt-in market research panel and respondents were randomly selected among those likely to qualify for eligibility. Eligibility criteria were Texas residents, aged 18 years or older, and could read and respond to a survey in English. Participants were recruited online from June to July 2022. There was a total of 1213 respondents, of which, 1089 presented complete/verified responses (i.e., answered the quality item correctly, met the minimum threshold for time to complete) resulting in a 90% completion rate. 

A total of 1020 completed survey responses were made available by Qualtrics after backend data processing. The 24 cases that responded with “don’t know” or “prefer not to answer” to vaccination status were removed as this was the primary outcome of the present study, thereby resulting in a total of 996 cases. Additionally, five cases with “don’t know/prefer not to answer” for education level were filtered, and those with a gender identity other than “man” or “woman” were not included due to the small cell size (*n* = 6), resulting in a final analysis sample of *n* = 985.

### 2.2. Measures

The measures selected for this survey were derived from a Common Survey instrument developed by the NIH’s Community Engagement Alliance (CEAL) Against COVID-19 Disparities Assessment and Evaluation Workgroup. Common measures were developed to permit similar assessment strategies across states involved in the CEAL initiative. The primary outcome of this study was on COVID-19 vaccination status and included predictor variables related to demographics, COVID-19 infection/vaccine attitudes and beliefs, and challenges related to the COVID-19 pandemic.

#### 2.2.1. Vaccination Status

The main outcome of interest was COVID-19 vaccination status. Respondents were asked: “Have you received at least one dose of the COVID-19 vaccine?”. Response options included: “No, have not gotten the vaccine”, “Yes, received the first dose of two-dose vaccine”, “Yes, received one dose of the vaccine”. “Yes, received both doses of the two-dose vaccine”, “Yes, got both doses of two-dose vaccine and a booster”, and “Yes, received one dose of the vaccine and a booster”. Vaccination status was collapsed into three categories: unvaccinated, partially vaccinated (at least one dose, but no booster), or fully vaccinated (received the vaccine and a booster shot).

#### 2.2.2. Demographics

Respondents provided their self-reported race, Hispanic ethnicity, age, education level, and gender identity (see Results for response categories).

#### 2.2.3. COVID-19 Infection and Vaccine Attitudes/Beliefs

The survey included questions about confidence in the United States Food and Drug Administration (FDA) and attitudes related to fear of/conscientiousness about COVID-19 infection. Respondents were asked, “how much do you trust the (FDA) to ensure the COVID-19 vaccine is safe for the public?” Response options were “do not trust”, “somewhat trust”, “mostly trust” or “fully trust.” Participants were also asked to rate how likely it was for the following to occur “in the next 6 months”: “I will get COVID-19”, “if I get COVID-19, I will have to go to the hospital”, and “if I get COVID-19, I will make someone else sick,” rated on a numeric scale from 1 (“not at all likely”) to 6 (“already happened”, with 5 indicating “very likely”).

#### 2.2.4. Challenges Related to COVID-19 Pandemic 

Participants were asked about the challenges they may have faced due to the COVID-19 pandemic, regardless of whether they were infected or not. Respondents were asked if they faced challenges getting the healthcare that they needed, having a place to live, getting enough food to eat, having clean drinking water, obtaining necessary medications, having access to transportation (“getting where I need to go”), and taking care of children or other dependents in their care. Respondents rated each as either, not a challenge, a minor challenge, or a major challenge. Responses were collapsed into binary yes/no responses for analysis, with “minor challenge” and “major challenge” combined into one response.

### 2.3. Data Analysis

Descriptive statistics for the variables used in this analysis were first presented overall and stratified by vaccination status. Counts and percentages were computed for categorical variables, while the means and standard deviations (SDs) were calculated for the continuous variables. Multivariable multinomial regression was used to determine the variables that were associated with the likelihood of belonging to different vaccination status groups. The multinomial model included the three-category vaccination status outcome as the dependent variable with education, race, gender identity, age, trust in the FDA to ensure a safe COVID-19 vaccine, fear of/conscientiousness about COVID-19, and challenges related to the COVID-19 pandemic as independent variables. 

## 3. Results

### 3.1. Descriptive Statistics

Table 1 provides descriptive statistics across the variables included in the analysis, both overall and stratified by vaccination status. A total of 29 individuals identified as Native Hawaiian/Pacific Islander, American Indian or Alaska Native, or multiracial; these individuals were combined into one category due to small individual group sizes, labeled “Other”.

Overall, the sample was 40% White non-Hispanic, 39.5% Hispanic, 11.8% Black non-Hispanic, 5% Asian, and 2.9% other. Most (54.2%) of the sample reported an education level of some high school, high school degree, or some college, whereas 45.8% had some higher degree. The average age of survey respondents was 42.7 years old (SD = 17.2). Approximately 9.5% (*n* = 94) were born outside of the US.

When stratifying by vaccination status, a few notable patterns emerged. A vast majority of Asian individuals were at least partially vaccinated (38.8%) or fully boosted (53.1%); other racial groups had between 24.7% and 44.8% unvaccinated persons. A plurality of White non-Hispanic and Hispanic/Latinx respondents was boosted (48.8% and 39.1%, respectively), whereas the largest vaccination status group among Black non-Hispanic individuals was partially vaccinated (36.2%). The largest vaccination group among those in the “Other” race category was unvaccinated (44.8%). Non-US-born respondents had high rates of vaccination, with 10% unvaccinated, 37% partially vaccinated, and 52% boosted. Additionally, boosted individuals tended to be older (mean age = 47.7 years) compared to unvaccinated (mean = 40.0 years) and partially vaccinated (mean = 38.3 years). Regarding education level, those without a higher degree had the largest share of unvaccinated individuals (36.5%), with only 30.5% fully boosted. Conversely, a majority of individuals with a bachelor’s or graduate degree were fully boosted (60.7% and 65.5%, respectively), and these education levels had small groups of unvaccinated individuals (12.1% and 9.5%, respectively). The non-US-born respondents tended to have higher levels of education.

### 3.2. Multinomial Regression

The multinomial regression model included three possible direct comparisons for computing odds ratio estimates: unvaccinated vs. partially vaccinated, unvaccinated vs. fully boosted, and partially vaccinated vs. fully boosted. The subsections below highlight the key findings for each comparison. Table 2 contains the full results for all comparisons in the multinomial model.

#### 3.2.1. Unvaccinated vs. Partially Vaccinated

In the regression model, Asian Americans were more likely to be partially vaccinated than unvaccinated compared to White non-Hispanic individuals (OR = 5.56, 95% CI 1.63, 18.94). Hispanic respondents were also more likely to be vaccinated than unvaccinated when compared to White non-Hispanic respondents (OR = 1.72, 95% CI 1.09, 2.71). We found no significant differences between White and Black Texans or those reporting an “other” race regarding vaccination status. Obtaining a higher education degree resulted in higher odds of vaccination compared to being unvaccinated (Table 2). There were no differences between those who reported a gender identity of man or woman, and there was not a significant effect of age when comparing unvaccinated to partially vaccinated status. Additionally, increasingly higher levels of trust compared to no trust at all in the FDA to ensure a safe vaccine were associated with an increasingly higher likelihood of being partially vaccinated compared to unvaccinated (Table 2). For example, those who reported fully trusting the FDA were 7.92 times more likely to be vaccinated (95% CI 3.97, 15.80) compared to those who said they did not trust the FDA at all. Regarding fear of COVID-19, individuals who stated more fear of becoming infected or being hospitalized over the next 6 months were somewhat more likely to be partially vaccinated than unvaccinated. Rating a higher likelihood of infecting others with COVID-19 was not associated with partial vaccination compared to being unvaccinated. Only one COVID-19 pandemic-related challenge was associated with being partially vaccinated: those who said having clean drinking water was a challenge were less likely to be vaccinated.

#### 3.2.2. Unvaccinated vs. Fully Boosted

As in the comparison between unvaccinated and partially vaccinated status, Asian individuals were significantly more likely to be boosted compared to White non-Hispanic individuals (OR = 5.47, 95% CI 1.52, 19.61). Similarly, obtaining a higher degree was also strongly associated with a higher likelihood of being fully vaccinated compared to unvaccinated (Table 2). Again, no gender differences were found, although an increase in age was significantly associated with an increase in the likelihood of being boosted rather than unvaccinated. Trusting the FDA once again conferred higher odds of being boosted, and the increase in the odds was much larger for each education level compared to the odds of being partially vaccinated vs. unvaccinated (Table 2). However, none of the fear of COVID-19 questions was associated with any changes in the likelihood of being boosted compared to unvaccinated. Regarding the challenges faced, those who reported having difficulty with a place to live were more than twice as likely to be boosted than unvaccinated, and those reporting having a hard time with transportation were less likely to be boosted.

#### 3.2.3. Partially Vaccinated vs. Fully Boosted

There were no differences among racial groups in the likelihood of being partially vaccinated vs. boosted in comparison to the reference group of being non-Hispanic White. Obtaining an associate degree did not result in different odds of being partially vaccinated vs. boosted compared to not having any higher degree. Obtaining a bachelor’s or graduate degree was associated with a significant increase in the likelihood of being boosted compared to being partially vaccinated (Table 2). As before, no gender differences were found in vaccination status. Older individuals were significantly more likely to be boosted than partially vaccinated. Increasing trust in the FDA, once again, was associated with higher odds of being boosted compared to being only partially vaccinated. Only those who reported a greater chance of infecting others with COVID-19 in the next 6 months were more likely to be boosted compared to those partially vaccinated. Finally, two challenges faced during the pandemic were associated with differences in being fully boosted vs. partially vaccinated: those who reported having a place to live as a challenge were more likely to be boosted, and those reporting having enough food to eat as a challenge were significantly less likely to be boosted.

## 4. Discussion

This population-based survey intended to identify correlates of COVID-19 vaccination (completely vaccinated, partially vaccinated, and not vaccinated) to discover potential areas for improvement of vaccination rates throughout the state of Texas. Reporting trust in the FDA to ensure a safe vaccine had the strongest impact on vaccination, with increasing levels of trust associated with much higher odds of being fully vaccinated compared to partially vaccinated, as well as higher odds of being partially or fully vaccinated compared to unvaccinated. Our results also showed that Asian respondents were much more likely to be vaccinated or boosted compared to White respondents, and Hispanic individuals were more likely to be partially vaccinated as opposed to unvaccinated than White non-Hispanic individuals. Higher education levels, fear of getting COVID-19 or spreading it to others, and reporting housing insecurity as a challenge brought on by the COVID-19 pandemic were also associated with greater odds of vaccination. Additionally, psychosocial factors, including worrying about basic needs, may contribute to COVID-19 vaccination. Specifically, we found that worrying about not having access to clean drinking water or transportation as a result of the pandemic was associated with a lower likelihood of obtaining a vaccination. 

Results showed that Asian and Hispanic/Latinx respondents had higher vaccination rates than White non-Hispanic and Black non-Hispanic individuals, while the “Other” racial groups were not different from White non-Hispanics in terms of vaccination likelihood. Current data from the National Immunization Survey Adult COVID Module showed that Asians (98.0%), Black non-Hispanics (90.1%), and Hispanics (87.7%) have received at least one dose of the COVID-19 vaccine, compared with 86.4% of White non-Hispanic adults [17]. These findings are inconsistent with previous studies, which report that Asians and White non-Hispanics have higher vaccination rates compared to Hispanic, Black non-Hispanic, and other racial minority groups [18,19]. Prior to availability of the COVID-19 vaccine, studies showed that Asians and Hispanics/Latinos reported greater intentions to get the vaccine and Black non-Hispanic/non-Latinx were more hesitant to get the vaccine if a vaccine was approved [20,21]. This pattern is still evident as studies conducted during the post-vaccine period show that Black non-Hispanics were less willing to get the COVID-19 vaccine compared to other racial and ethnic groups [22,23]. There are also known trends in racial and ethnic differences regarding general adult vaccinations, with minority populations having lower vaccination rates [24,25]. Our findings suggest the need to further investigate the factors that influence the decision to get vaccinated against COVID-19, especially with booster doses, among the different racial and ethnic groups. Distrust of the medical system or healthcare providers can influence the willingness to get the vaccine [26,27,28], which emphasizes the need for strategies that will help improve provider–patient relationships or strengthen community engagement activities by healthcare educators and professionals. Furthermore, conversations surrounding COVID-19 vaccination should be tailored to address information gaps. 

Our study findings also reported that older individuals were more likely to be boosted. A study among adults between the ages of 18 and 39 years old found that young adults (18–24 years) were undecided about the vaccine and had lower COVID-19 vaccination rates compared with older adults within the 35–39-year age range [29]. Young adults may have a perceived low vulnerability to COVID-19, which can influence their motivation to get the vaccine. Moreover, recommendations have highlighted the need for older age groups, especially those in nursing and assisted living facilities, to receive the vaccine due to their increased susceptibility to COVID-19 [30,31]. Additional studies should be initiated to explore strategies that may increase COVID-19 vaccination rates among younger age groups. Furthermore, our results showed that more than a third of respondents with either a high school degree or some college were not vaccinated, which is consistent with existing research that individuals with lower educational levels showed higher hesitancy towards COVID-19 vaccination and reported lower vaccination rates [22,32]. Individuals with lower education levels may have limited science health literacy, influencing confidence in the safety and effectiveness of the vaccine. This emphasizes the need to examine information gaps concerning the COVID-19 vaccine to help improve health literacy among subpopulations.

Beyond demographics, our study found that those with greater trust in the FDA to ensure the safety of COVID-19 vaccines were more likely to receive the vaccine. One possible explanation for this positive association could be that the FDA has a continuous safety monitoring system in place for COVID-19 vaccines and closely monitors potential adverse events of interest (AEI) associated with the COVID-19 vaccine, such as near real-time surveillance [33]. The surveillance findings are available to the public, and the rate of adverse events has remained low, which might increase participants’ confidence in receiving the COVID-19 vaccine. Even though our data demonstrate a positive association between the FDA’s safety assurance of the COVID-19 vaccine and vaccine uptake, it is difficult to conclude exactly how this assurance reaches individuals. According to a prior study, low uptake of the COVID-19 vaccine was found to be associated with concerns regarding the safety and efficacy of the vaccine, misinformation, and scientific ambiguity [34,35]. Developing messages that educate the public on the safety and efficacy of COVID vaccines is important to overcome vaccine hesitancy and mistrust, thereby increasing vaccination rates. By providing accurate and reliable information on vaccination through a variety of sources, including social media, healthcare providers, and community organizations, the public is more likely to understand the benefits of getting vaccinated and make informed decisions regarding the COVID-19 vaccine. Additionally, utilizing multiple channels can help reach diverse populations and ensure that the information is accessible to all.

Moreover, our findings indicate that individual fear/conscientiousness about getting COVID-19 or hospitalization is positively associated with getting the vaccine among the participants who were partially vaccinated compared to the unvaccinated; however, this association was not present when comparing the boosted with the unvaccinated or the boosted with the vaccinated. Possible causes for this positive relationship of being partially vaccinated could be that the participants’ belief that any dose of the COVID-19 vaccine will offer sufficient immunity. Even though our data demonstrate a positive association between the individual’s fear of being infected or hospitalized and being partially vaccinated, we found that fear of infecting others was only associated with being boosted compared to being partially vaccinated and did not contribute to differences in the odds of being unvaccinated vs. vaccinated. Social responsibility may contribute to this association. Participants who are caregivers, healthcare professionals, or members of community service organizations, for example, may be more likely (or required by employers) to obtain the booster dose out of fear of infecting other people. However, it is possible that we saw no difference between the unvaccinated and vaccinated regarding fear of infecting others due to the belief that any vaccination confers protection against infecting others. 

Analysis performed on the data regarding the challenges presented by the COVID-19 pandemic resulted in inconsistent findings for this study. Overall, the challenges that participants faced due to the pandemic that had an impact on vaccination status (in one direction or another) were having clean healthy water, having a place to live, access to required healthcare services and medications, having enough food to eat, access to transportation, and being able to take care of children or others throughout the pandemic. Individuals who reported not having the same access to transportation as they did prior to the pandemic were less likely to be boosted compared to unvaccinated. In this case, lack of transportation itself could prevent people from receiving the vaccination. Another example is individuals who were worried about having water and food being less likely to be partially vaccinated (water) or less likely to be fully vaccinated (food). On the other hand, the challenges could have also motivated individuals to receive the vaccine. For example, a positive impact was seen in individuals facing challenges with housing quality and security, as they were much more likely to be boosted. Here, the inclination to receive the vaccine may be motivated by hopes of returning to “normalcy,” or their pre-pandemic life. Thus, vaccination status can differ based on how challenges faced during the pandemic were perceived, leading to a lack of consistent findings on the impact of the challenges on vaccination.

The National Institutes of Health has invested considerable resources in states in order to facilitate community engagement and community-engaged research related to COVID-19 health disparities, which included vaccination uptake [16]. Texas has been involved in the Community Engagement Alliance (CEAL) Against COVID-19 Disparities since September 2020 through the Texas CEAL Consortium, which is composed of academic–community partnerships in 16 counties in Texas [36]. These partnerships focus on alleviating COVID-19 health disparities among Black, Hispanic/Latinx, and Asian populations in Texas. The findings from this study demonstrate that disparities in COVID-19 vaccination status were not present for some historically marginalized racial and ethnic groups. Instead, beliefs regarding trust in government entities and COVID-19 fear were salient factors related to vaccination status. As efforts continue in the state to address COVID-19 and the consequences of COVID-19, recognizing the messages that may resonate with individuals who are unvaccinated or not up to date on COVID-19 vaccination boosters are needed by trusted community messengers. 

This study does not withstand limitations. First, the study sample was from an online panel of English speakers who reported living in Texas. Selection bias may be present for those who opted to participate in the study compared to non-respondents. Similarly, the findings cannot be generalized to all residents in Texas given the inclusion criteria and sampling frame. We were also unable to describe the communities in which participants reside (e.g., urban, rural), which may contribute to the access to COVID-19 vaccination. Secondly, this was a cross-sectional study design, and we cannot establish temporality between COVID-19 vaccination status and the challenges experienced due to the COVID-19 pandemic. Finally, COVID-19 vaccination status was self-reported and could not be independently verified based on the study’s methodology. There may be a non-differential misclassification of vaccination status, as well as potential social desirability bias in reporting COVID-19 vaccination and other psychosocial variables. 

## 5. Conclusions

Our study shows that race/ethnicity, education, trust, and perception about the COVID-19 vaccine are associated with COVID-19 vaccination status. These findings emphasize the need to further investigate the interaction between individual and contextual factors in improving COVID-19 vaccination rates, especially among vulnerable or disadvantaged populations. Furthermore, more studies should explore the facilitators and barriers to accessing the vaccine among racial/ethnic and age groups. This can help tailor appropriate community strategies to meet the needs of populations. In addition, different strategies may need to be initiated for individuals who are partially vaccinated versus unvaccinated. Public health messages should be tailored to address questions on the safety and health implications of the vaccine to improve trust and confidence in the vaccine.

## Figures and Tables

**Table 1:** Descriptive statistics of adults in Texas sample, June–July 2022 (*n* = 985).

Variable	Category	Total *n* (Column Wise%)	Unvaccinated *n* (Row Wise%)	Vaccinated *n* (Row Wise%)	Boosted *n* (%)
Vaccination status	-	985 (100)	261 (26.5)	308 (31.3)	416 (42.2)
Race/ Ethnicity	White non-Hispanic	402 (40.8)	110 (27.4)	96 (23.9)	196 (48.8)
	Black/African American non-Hispanic	116 (11.8)	38 (32.8)	42 (36.2)	36 (31.0)
	Hispanic/Latinx	389 (39.5)	96 (24.7)	141 (36.2)	152 (39.1)
	Asian	49 (5.0)	4 (8.2)	19 (38.8)	26 (53.1)
	Other	29 (2.9)	13 (44.8)	10 (34.5)	6 (20.7)
Education	High School degree and some college	534 (54.2)	195 (36.5)	176 (33.0)	163 (30.5)
	Associate degree	121 (12.3)	29 (24.0)	45 (37.2)	47 (38.8)
	Bachelor’s degree	214 (21.7)	26 (12.1)	58 (27.1)	130 (60.7)
	Graduate degree	116 (11.8)	11 (9.5)	29 (25.0)	76 (65.5)
Gender identity	Man	405 (41.1)	91 (22.5)	115 (28.4)	199 (49.1)
	Woman	580 (58.9)	170 (29.3)	193 (33.3)	217 (37.4)
Trust FDA to ensure a safe vaccine?	Not at all	159 (16.1)	96 (60.4)	44 (27.7)	19 (11.9)
	Somewhat trust	263 (26.7)	99 (37.6)	85 (32.3)	79 (30.0)
	Mostly trust	337 (34.2)	50 (14.8)	119 (35.3)	168 (49.9)
	Fully trust	226 (22.9)	16 (7.1)	60 (26.5)	150 (66.4)
Challenges					
getting healthcare	Yes	336 (34.1)	84 (25.0)	110 (32.7)	142 (42.3)
	No	649 (65.9)	177 (27.3)	198 (30.5)	274 (42.2)
Having a place to live	Yes	246 (25.0)	69 (28.0)	78 (31.7)	99 (40.2)
	No	739 (75.0)	192 (26.0)	230 (31.1)	317 (42.9)
Getting enough food	Yes	344 (34.9)	104 (30.2)	123 (35.8)	117 (34.0)
	No	641 (65.1)	157 (24.5)	185 (28.9)	299 (46.6)
Having clean water to drink	Yes	179 (18.2)	55 (30.7)	49 (27.4)	75 (41.9)
	No	806 (81.8)	206 (25.6)	259 (32.1)	341 (42.3)
Getting medications	Yes	290 (29.4)	78 (26.9)	93 (32.1)	119 (41.0)
	No	695 (70.6)	183 (26.3)	215 (30.9)	297 (42.7)
Getting where I need to go	Yes	341 (34.6)	105 (30.8)	118 (34.6)	118 (34.6)
	No	644 (65.4)	156 (24.2)	190 (29.5)	298 (46.3)
Taking care of my children/dependents	Yes	228 (23.1)	59 (25.9)	77 (33.8)	92 (40.3)
	No	757 (76.9)	202 (26.7)	231 (30.5)	324 (42.8)
**Continuous Variables**		**Overall Mean (SD)**	**Unvaccinated Mean (SD)**	**Vaccinated Mean (SD)**	**Boosted Mean (SD)**
Fear of/conscientiousness about COVID-19	How likely are you to get infected?	2.31 (1.51)	2 (1.46)	2.34 (1.57)	2.49 (1.47)
	If infected, how likely hospitalized?	2.19 (1.41)	1.91 (1.31)	2.24 (1.46)	2.34 (1.42)
	If infected, how likely to infect others?	2.90 (1.59)	2.72 (1.68)	2.88 (1.61)	3.02 (1.52)
Age		42.7 (17.2)	40.0 (15.6)	38.3 (14.0)	47.7 (18.9)

**Table 2:** Odds of COVID-19 vaccination status among a sample of adults in Texas, June–July 2022 (*n* = 985).

	Vaccination Status Comparison vs. Reference Group *:	Vaccinated vs. No	Boosted vs. No	Boosted vs. Vaccinated
Variable	Response	OR (95% CI)	OR (95% CI)	OR (95% CI)
Race/ ethnicity	White non-Hispanic	1 [reference]	1 [reference]	1 [reference]
	Black/African American non-Hispanic	1.36 (0.75, 2.47)	0.96 (0.50, 1.86)	0.70 (0.40, 1.25)
	Hispanic/Latinx	**1.72 (1.09, 2.71)**	1.57 (0.97, 2.53)	0.91 (0.61, 1.37)
	Asian	**5.56 (1.63, 18.94)**	**5.47 (1.52, 19.61)**	0.98 (0.47, 2.04)
	Other	0.86 (0.33, 2.22)	0.36 (0.11, 1.16)	0.42 (0.14, 1.28)
Education	High School degree and some college	1 [reference]	1 [reference]	1 [reference]
	Associate degree	**2.17 (1.25, 3.79)**	**2.46 (1.35, 4.50)**	1.13 (0.68, 1.88)
	Bachelor’s degree	**2.94 (1.68, 5.15)**	**6.37 (3.63, 11.17)**	**2.17 (1.43, 3.28)**
	Graduate degree	**3.01 (1.37, 6.63)**	**6.18 (2.86, 13.36)**	**2.05 (1.21, 3.49)**
Gender identity	Man	1 [reference]	1 [reference]	1 [reference]
	Woman	0.96 (0.65, 1.42)	0.76 (0.51, 1.14)	0.79 (0.56, 1.11)
Trust FDA to ensure a safe vaccine?	Not at all	1.0 (reference)	1 [reference]	1 [reference]
	Somewhat trust	**2.03 (1.24, 3.33)**	**6.06 (3.19, 11.51)**	**2.98 (1.55, 5.75)**
	Mostly trust	**5.46 (3.23, 9.22)**	**25.31 (13.07, 48.99)**	**4.64 (2.47, 8.71)**
	Fully trust	**7.92 (3.97, 15.80)**	**54.14 (24.86, 117.93)**	**6.84 (3.53, 13.23)**
Fear of/conscientiousness about COVID-19	How likely are you to get infected?	**1.15 (1.00, 1.32)**	1.11 (0.96, 1.28)	0.96 (0.86, 1.09)
	If infected, how likely hospitalized?	**1.17 (1.01, 1.36)**	1.15 (0.98, 1.35)	0.98 (0.86, 1.12)
	If infected, how likely to infect others?	0.92 (0.80, 1.05)	1.04 (0.91, 1.20)	**1.14 (1.01, 1.28)**
Age	continuous	1.00 (0.99, 1.01)	**1.03 (1.02, 1.05)**	**1.03 (1.02, 1.05)**
Challenges	No	1 [reference]	1 [reference]	1 [reference]
Getting healthcare	Yes	1.04 (0.66, 1.64)	1.08 (0.66, 1.78)	1.05 (0.69, 1.58)
Having a place to live	Yes	1.19 (0.69, 2.06)	**2.09 (1.14, 3.84)**	**1.75 (1.04, 2.95)**
Getting enough food	Yes	1.14 (0.67, 1.92)	0.57 (0.32, 1.04)	**0.51 (0.31, 0.83)**
Having clean water to drink	Yes	**0.40 (0.22, 0.74)**	0.66 (0.34, 1.28)	1.65 (0.93, 2.96)
Getting medications	Yes	1.08 (0.65, 1.80)	1.41 (0.81, 2.48)	1.31 (0.81, 2.11)
Getting where I need to go	Yes	0.79 (0.48, 1.29)	**0.51 (0.29, 0.87)**	0.64 (0.41, 1.02)
Taking care of my children/dependents	Yes	1.15 (0.67, 1.97)	0.97 (0.53, 1.77)	0.85 (0.51, 1.42)

* Multinomial models comparing likelihood-adjusted odds ratios of vaccination status across categorical and continuous independent variables. The ORs compare the likelihood of being in the first vaccination category in reference to the second category for each column when moving from the reference category of each independent variable into the other categories., e.g., for the trust variable, the first column of ORs represents the odds of being partially vaccinated compared to being unvaccinated”, and the “somewhat trust” OR is the adjusted odds of responding vaccinated vs. unvaccinated for someone who somewhat trusts the FDA compared to not at all trusting the FDA. ORs that do not contain 1.00 in the interval are statistically significant at the level.

## Data Availability

Please contact the authors for data availability.

## References

[1] The Texas Department of State Health Services Texas COVID-19 Cases Dashboard. https://txdshsea.maps.arcgis.com/apps/dashboards/4ae43eefd0f641d59d35c3df82ee59cc.

[2] Ojinnaka C.O., Adepoju O.E., Burgess A.V., Woodard L. (2021). Factors Associated with COVID-Related Mortality: The Case of Texas. J. Racial Ethn. Health Disparities.

[3] Centers for Disease Control and Prevention How to Protect Yourself and Others. https://www.cdc.gov/coronavirus/2019-ncov/prevent-getting-sick/prevention.html.

[4] Abbott G. (2021). Governor Abbott Lifts Mask Mandate, Opens Texas 100 Percent.

[5] Allen R. (2021). The Texas Tribune. https://www.texastribune.org/2021/10/11/texas-greg-abbott-covid-19-vaccine-mandate.

[6] Centers for Disease Control and Prevention (2023). Stay Up to Date with COVID-19 Vaccines Including Boosters.

[7] Centers for Disease Control and Prevention (2023). COVID-19 Vaccinations in the United States. https://covid.cdc.gov/covid-data-tracker/#vaccinations_vacc-people-additional-dose-totalpop.

[8] Sah P., Moghadas S.M., Vilches T.N., Shoukat A., Singer B.H., Hotez P.J., Schneider E.C., Galvani A.P. (2021). Implications of suboptimal COVID-19 vaccination coverage in Florida and Texas. Lancet Infect. Dis..

[9] National Conference of State Legislatures HPV Vaccine State Legislation and Statutes. https://www.ncsl.org/research/health/hpv-vaccine-state-legislation-and-statutes#:~:text=As%20of%20April%202020%2C%20at,vaccine%20starting%20July%201%2C%202020.

[10] (2007). H.B.1098—(2007); Legislature of the State of Texas, Austin, TX, USA. https://capitol.texas.gov/tlodocs/80R/billtext/html/HB01098F.htm.

[11] Olive J.K., Hotez P.J., Damania A., Nolan M.S. (2018). The state of the antivaccine movement in the United States: A focused examination of nonmedical exemptions in states and counties. PLoS Med..

[12] Morrison M., Castro L.A., Ancel Meyers L. (2020). Conscientious vaccination exemptions in kindergarten to eighth-grade children across Texas schools from 2012 to 2018: A regression analysis. PLoS Med..

[13] Litaker J.R., Tamez N., Lopez Bray C., Durkalski W., Taylor R. (2022). Sociodemographic Factors Associated with Vaccine Hesitancy in Central Texas Immediately Prior to COVID-19 Vaccine Availability. Int. J. Environ. Res. Public Health.

[14] Berenson A.B., Chang M., Hirth J.M., Kanukurthy M. (2021). Intent to get vaccinated against COVID-19 among reproductive-aged women in Texas. Hum. Vaccines Immunother..

[15] Amundson C.J., Sias J.J., Frietze G.A. (2022). Perceptions of COVID-19 vaccines in a predominantly Hispanic patient population from the Texas-Mexico border. J. Am. Pharm. Assoc..

[16] Seguin-Fowler R.A., Amos C., Beech B.M., Ferrer R.L., McNeill L., Opusunju J.J., Spence E., Thompson E.L., Torres-Hostos L.R., Vishwanatha J.K. (2022). The Texas Community-Engagement Research Alliance Against COVID-19 in Disproportionately Affected Communities (TX CEAL) Consortium. J. Clin. Transl. Sci..

[17] Centers for Disease Control and Prevention Trends in Demographic Characteristics of People Receiving COVID-19 Vaccinations in the United States. https://covid.cdc.gov/covid-data-tracker/#vaccination-demographics-trends.

[18] Williams A.M., Clayton H.B., Singleton J.A. (2022). Racial and Ethnic Disparities in COVID-19 Vaccination Coverage: The Contribution of Socioeconomic and Demographic Factors. Am. J. Prev. Med..

[19] Zhang W., Wu Y.Y., Wu B. (2022). Racial/Ethnic Disparities in Getting COVID-19 Vaccine: Do Age, Gender, and Education Matter?. Health Equity.

[20] Reiter P.L., Pennell M.L., Katz M.L. (2020). Acceptability of a COVID-19 vaccine among adults in the United States: How many people would get vaccinated?. Vaccine.

[21] Willis D.E., Andersen J.A., Bryant-Moore K., Selig J.P., Long C.R., Felix H.C., Curran G.M., McElfish P.A. (2021). COVID-19 vaccine hesitancy: Race/ethnicity, trust, and fear. Clin. Transl. Sci..

[22] Adzrago D., Sulley S., Ormiston C.K., Mamudu L., Williams F. (2022). Differences in the Perceived Likelihood of Receiving COVID-19 Vaccine. Int. J. Environ. Res. Public Health.

[23] Kricorian K., Turner K. (2021). COVID-19 Vaccine Acceptance and Beliefs among Black and Hispanic Americans. PLoS ONE.

[24] Lu P.-j., O’Halloran A., Williams W.W., Lindley M.C., Farrall S., Bridges C.B. (2015). Racial and ethnic disparities in vaccination coverage among adult populations in the U.S. Vaccine.

[25] Kawai K., Kawai A.T. (2021). Racial/Ethnic and Socioeconomic Disparities in Adult Vaccination Coverage. Am. J. Prev. Med..

[26] Bagasra A.B., Doan S., Allen C.T. (2021). Racial differences in institutional trust and COVID-19 vaccine hesitancy and refusal. BMC Public Health.

[27] Daly M., Robinson E. (2021). Willingness to Vaccinate Against COVID-19 in the U.S.: Representative Longitudinal Evidence From April to October 2020. Am. J. Prev. Med..

[28] Niño M., Harris C., Drawve G., Fitzpatrick K.M. (2021). Race and ethnicity, gender, and age on perceived threats and fear of COVID-19: Evidence from two national data sources. SSM Popul. Health.

[29] Baack B.N., Abad N., Yankey D., Kahn K.E., Razzaghi H., Brookmeyer K., Kolis J., Wilhelm E., Nguyen K.H., Singleton J.A. (2021). COVID-19 Vaccination Coverage and Intent Among Adults Aged 18–39 Years—United States, March–May 2021. MMWR Morb. Mortal. Wkly. Rep..

[30] (2020). Centers for Disease Control and Prevention. The Advisory Committee on Immunization Practices’ Interim Recommendation for Allocating Initial Supplies of COVID-19 Vaccine—United States. https://www.cdc.gov/mmwr/volumes/69/wr/mm6949e1.htm?s_cid=mm6949e1_w.

[31] Centers for Disease Control and Prevention COVID-19 Recommendations for Older Adults. https://www.cdc.gov/aging/covid19/index.html.

[32] Badr H., Zhang X., Oluyomi A., Woodard L.D., Adepoju O.E., Raza S.A., Amos C.I. (2021). Overcoming COVID-19 Vaccine Hesitancy: Insights from an Online Population-Based Survey in the United States. Vaccines.

[33] Initial Results of Near Real-Time Safety Monitoring of COVID-19 Vaccines in Persons Aged 65 Years and Older. https://www.fda.gov/vaccines-blood-biologics/safety-availability-biologics/initial-results-near-real-time-safety-monitoring-covid-19-vaccines-persons-aged-65-years-and-older.

[34] Skafle I., Nordahl-Hansen A., Quintana D.S., Wynn R., Gabarron E. (2022). Misinformation About COVID-19 Vaccines on Social Media: Rapid Review. J. Med. Internet Res..

[35] Butler J.Z., Carson M., Rios-Fetchko F., Vargas R., Cabrera A., Gallegos-Castillo A., LeSarre M., Liao M., Woo K., Ellis R. (2022). COVID-19 vaccination readiness among multiple racial and ethnic groups in the San Francisco Bay Area: A qualitative analysis. PLoS ONE.

[36] Thompson E.L., Beech B.M., Ferrer R.L., McNeil L.H., Opusunju J.J., Seguin-Fowler R.A., Spence E.E., Torres-Hostos L., Amos C.I., Desai P. (2022). Implementation of the Texas Community-Engaged Statewide Consortium for the Prevention of COVID-19. Int. J. Environ. Res. Public Health.

